# Daily Energy Expenditure, Cardiorespiratory Fitness and Glycaemic Control in People with Type 1 Diabetes

**DOI:** 10.1371/journal.pone.0097534

**Published:** 2014-05-14

**Authors:** John Joseph Valletta, Andrew J. Chipperfield, Geraldine F. Clough, Christopher D. Byrne

**Affiliations:** 1 Human Development and Health, Faculty of Medicine, University of Southampton, Southampton, United Kingdom; 2 Bioengineering Science, Faculty of Engineering and the Environment, University of Southampton, Southampton, United Kingdom; Tecnologico de Monterrey, Mexico

## Abstract

**Objective:**

Encouraging daily physical activity improves cardiorespiratory fitness and many cardiovascular risk factors. However, increasing physical activity often creates a challenge for people with type 1 diabetes, because of difficulties maintaining euglycemia in the face of altered food intake and adjustments to insulin doses. Our aim was to examine the triangular relationship between glucose control measured by continuous glucose monitoring system (CGMS), objective measures of total daily energy expenditure (TEE) recorded by a multi-sensory monitoring device, and cardiorespiratory fitness (CRF), in free-living subjects with type 1 diabetes.

**Research Design and Methods:**

Twenty-three individuals (12 women) with type 1 diabetes who were free from micro- and macrovascular complications were recruited. TEE and glucose control were monitored simultaneously for up to 12 days, using a multi-sensory device and CGMS respectively. CRF was recorded as V02 max from a maximal treadmill test with the Bruce protocol.

**Results:**

Subjects (mean±SD) were aged 37±11 years, with BMI = 26.5±5.1 kg.m^−2^, HbA_1c_ = 7.7±1.3% (61±14 mmol/mol) and V02 max (ml.min^−1^.kg^−1^)  = 39.9±8.4 (range 22.4 – 58.6). TEE (36.3±5.5 kcal.kg^−1^.day^−1^) was strongly associated with CRF(39.9±8.4 ml.min^−1^.kg^−1^) independently of sex (r = 0.63, p<0.01). However, neither TEE (r = −0.20, p = 0.36) nor CRF (r = −0.20, p = 0.39; adjusted for sex), were significantly associated with mean glycaemia measured by CGMS.

**Conclusion:**

Higher levels of energy expenditure (due to a more active lifestyle) are associated with increased cardiorespiratory fitness, but not necessarily better glycaemic control. Since increased levels of energy expenditure *and* good glycaemic control are both needed to protect against diabetes-related complications our data suggest they need to be achieved independently.

## Introduction

The health and general well-being benefits of high levels of cardiorespiratory fitness (CRF) and physical activity are well documented in people with diabetes [1], [2]. Often, however, physical activity levels are sub-optimal in people with type 1 diabetes because of a fear of hypoglycaemia or low levels of CRF [3], [4]. Additionally, a further challenge facing people with type 1 diabetes is how best to ensure good glucose control in the presence of varying levels of food intake and insulin doses throughout the day. Too little or too much exogenous insulin causes erratic glucose control, particularly when individuals have varying levels of physical activity energy expenditure (PAEE). Such erratic control can have adverse affects on the individual's overall glycaemic control, thereby increasing the predisposition to vascular complications [5].

Most of the studies reported to date have studied the effect of specific exercise interventions (typically moderate intensity aerobic activities) on cardiovascular risk factors and glycaemic control. Physical activity interventions in people with type 1 diabetes have been linked to improved CRF [6]–[13], insulin sensitivity [6], [10], lipid profile [6]–[9], [13] and endothelium function [12], [14], but results investigating the association between levels of physical activity and glycaemic control have been contradictory [15]. Similar inconsistent results have been reported in observational studies that used questionnaires to quantify levels of physical activity. For example, long-term glucose control assessed by HbA_1c_ has been shown to be lower in people with type 1 diabetes engaging in increased levels of physical activity [16], [17], but Waden *et al.* [18] found that such an association was only present in women; while others found no correlation [19], and some even reported a positive relationship between HbA_1c_ and CRF [20]. Recently, Kennedy *et al* [21] concluded in their meta-analysis of 13 studies that there was no evidence of glycaemic benefit, measured by HbA_1c_, of exercise and further suggested that HbA_1c_ may not be a sufficiently sensitive indicator of glycaemic control.

It is plausible that these discrepant results between studies might have originated from imprecision in the measurements of energy expenditure, combined with a failure to accurately take account of potential confounders affecting the relationship between physical activity levels and glycaemic control (e.g. body fatness, energy intake and insulin dose). Energy expenditure quantified by validated questionnaires is only poorly-to-moderately accurate, with correlation coefficients ranging from r = 0.1 to 0.6 when compared to double labelled water (DLW) [22]. The accuracy of questionnaires in representing an individual's pattern of daily energy expenditure is therefore debatable. Due to advances in wearable sensing technologies and pattern recognition algorithms, light multi-sensory physical activity armbands can nowadays be used to obtain an objective measure of energy expenditure, and with a correlation coefficient of r = 0.86 with DLW [23], such devices offer clear advantages over questionnaires and/or simple accelerometers. Continuous glucose monitoring systems (CGMS) have lately given researchers a tool to obtain more precise estimates of short-term mean glycaemia and variability, which infrequent finger-prick measurements could not reveal. Using a multi-sensory physical activity armband combined with CGMS monitoring it is therefore possible to obtain estimates of 24 hr glucose control and energy expenditure in free-living people.

The aim of our pilot study was to examine the triangular relationship between glucose control measured by CGMS, levels of energy expenditure measured by a multi-sensory monitoring device, and cardiorespiratory fitness, in a heterogeneous group of free-living subjects with type 1 diabetes.

## Research Design and Methods

### Participants

**Figure 1:**
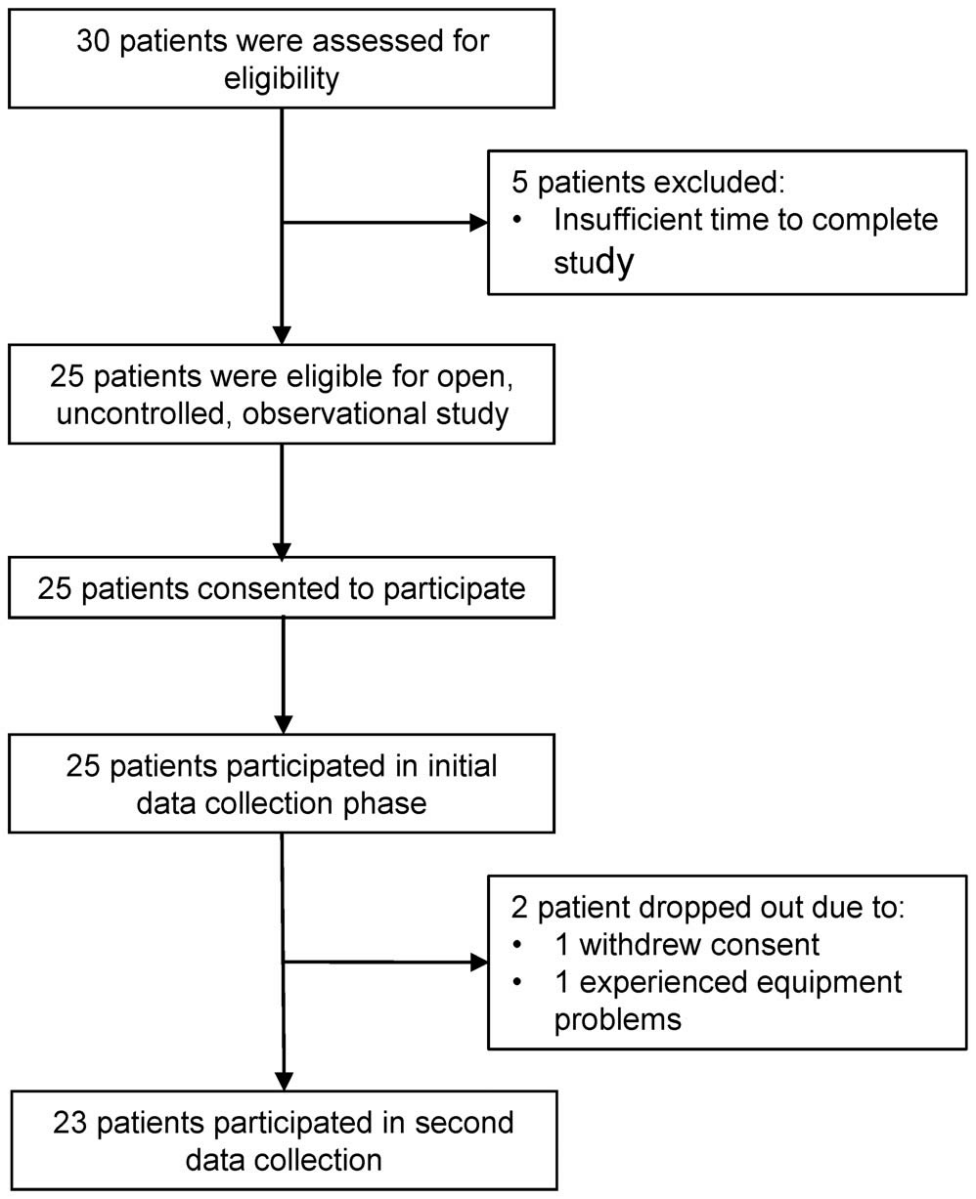
CONSORT flow diagram showing enrollment and retention of volunteers in the study of energy expenditure in type 1 diabetes.

Twenty-five complications-free people with type 1 diabetes were recruited in our open, non-randomised and observational study. The object of the study was to observe a varied cohort spanning the spectrum of energy expenditure from sedentary to more active individuals. Potential volunteers were identified from those attending the Diabetes Resource Centre at the Royal South Hants Hospital, Southampton, England. Subject selection criteria were non-stringent only requiring the participant to be on multiple insulin injections (and not on an insulin pump) and have no other acute problems. Invitation to participate was made directly by letter with a verbal explanation and patient information sheet before recruitment. Data from 23 (12 women) subjects are reported (one individual withdrew for personal reasons after recruitment and one individual had problems using the CGMS) as reported in the CONSORT diagram in Figure 1. The study protocol and TREND checklist are available as supporting information (Protocol S1 and Checklist S1). After giving signed consent, the participants undertook a number of clinical tests to determine total body fat and CRF. The participants were then issued with a multi-sensory physical activity monitory device and CGMS for free-living monitoring. Volunteers were recruited between 8^th^ July 2008 and 4^th^ December 2009 – there was no period of follow up.

### Ethics Statement

The ethical content of the study was reviewed by the Southampton and South West Hampshire Research Ethics Committee (LREC:07/H0502/134), and conforms to the principles outlined in the Declaration of Helsinki.

### Daily Energy Expenditure and Glucose Control in a Free-Living Environment

Daily energy expenditure was monitored by a SenseWear Pro2/3 armband (BodyMedia, PA, USA), while blood glucose (BG) was monitored by a Guardian® Real-Time CGMS (Medtronic MiniMed Inc., CA, USA). Both devices were issued to the participants after being taught how to use them, and instructed to contact the researcher should there be any problem in operating them. Particular emphasise was placed on abiding to the manufacturer's guidelines on calibrating the CGMS (2 h after sensor insertion, again after 6 h, then at least one calibration within every 12 h period). Frequent calibrations avoid issues with sensor drifts and thus improve the quality of the data. The multi-sensory physical activity armband and CGMS were worn simultaneously and continuously for up to 12 days, in order to obtain a snapshot of the individual's daily lifestyle and corresponding glucose control. The participants were free to partake in any activity and make any therapeutic decision which would affect their BG.

Average total daily, energy expenditure (TEE) in kcal kg^−1^ day^−1^ was used to quantify the volunteer's lifestyle and the Metabolic Equivalent (1 MET  =  1 kcal kg^−1^ hour^−1^) to compute themean daily percentage of time spent engaging in activitites of different intensity levels. The intensity categories used were sedentary (<2 METs), light (2–3 METs), moderate (3–6 METs) and vigorous (>6 METS) [24]. Short-term glycaemic control was quantified by the daily, mean (MBG), standard deviation (SD) and coefficient of variation (CV, standard deviation normalised by the mean) of BG. We also estimated the percentage of time spent at risk of hypoglycaemia (< 4 mmol/l), with normoglycaemia (4 – 11 mmol/l) and with hyperglycaemia (> 11 mmol/l). HbA_1c_ was measured to give an estimate of each individual's glycaemic control over the preceding 2–3 months prior to the research study. For all individuals between two and eight measurements of HbA_1c_ were available for the 2-year period prior to recruitment in the study.

The participants were also asked to keep a food and insulin diary throughout the monitoring period. Average daily carbohydrate intake was estimated by converting the self-reported meals into equivalent grams of carbohydrates using food databases, or for specific branded foods, the producer's stated nutritional facts. All of our volunteers were treated with multiple daily insulin injection (MDII) regimes. Insulin diaries were used to estimate the typical daily insulin dosage.

### Body Composition and Cardiorespiratory Fitness

Body composition was estimated by a dual X-ray absorptiometry (DEXA) scan using a Hologic Delfia W 4500 (Hologic, Bedford, USA). CRF was assessed by a maximal treadmill test. The Bruce protocol was chosen so as to challenge even the fittest people in our heterogeneous group, which spanned a wide spectrum of lifestyles, from fairly sedentary to very active people. The volunteer wore an air-tight mask (Hans Rudolph Inc., MO, USA), which had a gas sensor (Metalyzer 3B, Cortex Biophysik GmbH, Germany) attached to it, while heart rate was monitored using a Polar Electro T61 chest heart rate monitor (Polar Inc., Lake Success, NY, USA). The participant was asked to run on a treadmill (Woodway, GmbH, Germany) until exhaustion, unless they experienced chest pain or felt unwell. VO_2max_ was taken to be the final steady-state value for oxygen consumption. The Foster [25] and Pollack [26] equations, which are functions of the time spent on the treadmill under the Bruce protocol, were used for men and women respectively, to estimate CRF.

### Statistical Analysis

Free-living physical activity and CGMS measurements were averaged over 24 h periods and over the total number of days in order to obtain a single data point for each participant in the study. Univariate correlation analyses were performed using the Pearson correlation for normally distributed variables. Multiple linear regression modelling was undertaken to identify factors that were independently associated with CRF. All statistical analyses were performed on IBM® SPSS® Statistics 21. A value of p<0.05 was taken as statistically significant.

## Results

Table 1 shows the baseline characteristics of the 23 (12 women, age = 37±11 years) participants with type 1 diabetes recruited to the study. No participants had evidence of microvascular or macrovascular complications. Univariate associations between the three key study variables (MBG, TEE and CRF), and glycaemic control metrics (MBG, SD, CV, HbA_1c_) and lifestyle measures (TEE, CRF, total body fat, mean daily carbohydrate intake, mean daily insulin dosage) are summarised in Table 2.

**Table 1:** Baseline characteristics of of N = 23 participants with type 1 diabetes.

Age (years)	37±11
Diabetes Duration (years)	17±11
BMI (kg.m^−2^)	26.5±5.1
Total Body Fat (%)	27.9±9.2
Maximal Oxygen Consumption (ml.min^−1^.kg^−1^) (V02 max)	39.9±8.4
Total Cholesterol (mmol/l)	4.7±0.9
LDL Cholesterol (mmol/l)	2.8±0.9
HDL Cholesterol (mmol/l)	1.5±0.4 (1.0 – 2.8)
Triglyceride (mmol/l)	0.7±0.4 (0.4 – 2.7)
Fasting Glucose (mmol/l)	10.1±4.7
HbA_1c_ (%)	7.7±1.3
HbA_1c_ (mmol/mol)	61±14
Mean Daily Insulin Dosage (IU/day)	53±20
Mean Daily Carbohydrate Intake (g/day)	227±62
Mean Daily Energy Expenditure (kcal.kg^−1^.day^−1^)	36.3±5.5
Time Spent Sedentary (<2 METs) (%)	69.8±9.3
Time Spent in Light Activities (2 – 3 METs) (%)	17.5±6.3
Time Spent in Moderate Activities (3 – 6 METs) (%)	11.8±4.9
Time Spent in Vigorous Activities (> 6 METs) (%)	0.2±1.4 (0 – 4.8)
Time Spent Blood Glucose < 4 mmol/l (%)	3.5±8.6 (0 – 24.6)
Time Spent Blood Glucose 4 – 11 mmol/l (%)	72.3±16.0
Time Spent Blood Glucose > 11 mmol/l (%)	15.4±18.6 (0 – 76.3)

Data are mean ± SD for normally distributed variables and median ± IQR (range) for non-normally distributed variables. HDL cholesterol, triglyceride and time spent in vigorous activities, BG<4 and BG>11 were non-normally distributed.

**Table 2:** Partial correlation coefficients for daily mean blood glucose (MBG), average total daily energy expenditure (TEE) and cardiorespiratory fitness (CRF), with glycaemic control metrics and lifestyle measures.

	MBG (mmol/l)	TEE (kcal.kg^−1^.day^−1^)	CRF* (ml.min^−1^.kg^−1^)
**Glycaemic Control Metrics**			
MBG (mmol/l)	1.0	−0.20 (p = 0.36)	−0.20 (p = 0.39)
SD (mmol/l)	0.62 (p<0.01)	0.07 (p = 0.76)	−0.09 (p = 0.68)
CV (%)	−0.30 (p = 0.17)	0.23 (p = 0.28)	−0.003 (p = 0.99)
HbA_1c_ (%)	0.36 (p = 0.09)	−0.10 (p = 0.65)	−0.03 (p = 0.89)
**Lifestyle Measures**			
TEE (kcal.kg^−1^.day^−1^)	−0.20 (p = 0.36)	1.0	0.63 (p<0.01)
CRF (ml.min^−1^.kg^−1^)*	−0.20 (p = 0.39)	0.63 (p<0.01)	1.0
Total Body Fat (%)†	0.31 (p = 0.18)	−0.71 (p<0.001)	−0.78 (p<0.0001)
Mean Daily Carbohydrate Intake (g.day^−1^) ‡	−0.07 (p = 0.76)	0.69 (p<0.001)	0.18 (p = 0.44)
Mean Daily Insulin Dosage (IU.day^−1^) ‡	−0.14 (p = 0.54)	0.35 (p = 0.11)	−0.21 (p = 0.36)

* adjusted for sex

† adjusted for age and sex

‡ adjusted for body weight

MBG, which represents the average blood glucose concentration over a 24 hr period as measured by CGMS, was associated with SD (r = 0.62, p<0.01) but not with CV (r = −0.30, p = 0.17) of BG. MBG (short-term glycaemic control measure) and HbA_1c_ (long-term glycaemic control measure) were poorly correlated and did not achieve conventional statistical significance (r = 0.36, p = 0.09). No significant associations were found between MBG and lifestyle.

TEE is an objective measurement of the average energy expended by an individual during their daily routine. TEE was not associated with any of the glycaemic control metrics. However, TEE was strongly correlated with CRF (r = 0.63, p<0.01; adjusted for sex), percentage total body fat (r = −0.71, p<0.001; adjusted for age and sex) and average daily carbohydrate intake (r = 0.69, p<0.001; adjusted for body weight). Average daily carbohydrate was strongly associated with both levels of sedentary activity (r = −0.53, p<0.05; adjusted for body weight) and moderate activity (r = 0.53, p<0.05; adjusted for body weight). Carbohydrate intake was also associated with average daily insulin dosage (r = 0.58, p<0.01; adjusted for body weight), but not with glycaemic control metrics.

CRF quantifies the efficiency of the human body to transport and use oxygen during aerobic exercise and is therefore conceptually different from the energy expended by an individual during day-to-day activities. CRF was not correlated with glycaemic control metrics. It was however associated with diabetes duration (r = −0.43, p<0.05; adjusted for age), TEE (r = 0.63, p<0.01; adjusted for sex), percentage total body fat (r = −0.78, p<0.0001; adjusted for age and sex) and time spent at moderate activity levels (r = 0.58, p <0.05; adjusted for age and sex). The amount of time spent at high activity in this cohort is very small and thus we have inadequate power to assess relationships with high versus low intensity activity further.

Because CRF is a strong predictor of cardiovascular disease, the factors associated with CRF were further explored using multiple regression modelling. Age is a known non-modifiable factor affecting CRF. However in a model with CRF as the outcome and age and TEE as explanatory variables, age was found to be statistically non-significantly associated with CRF (β = 0.01, p = 0.94), whereas TEE was associated with CRF (β = 0.69, p = 0.001); and this regression model explained 47% of the variance in CRF (R^2^ = 0.47, p<0.01). In a second regression model with CRF as outcome, 56% of the variance in CRF was explained by TEE (β = 0.41, p = 0.054) and percentage body fat (β = −0.40, p = 0.059) (R^2^ = 0.56, p<0.001).

**Figure 2:**
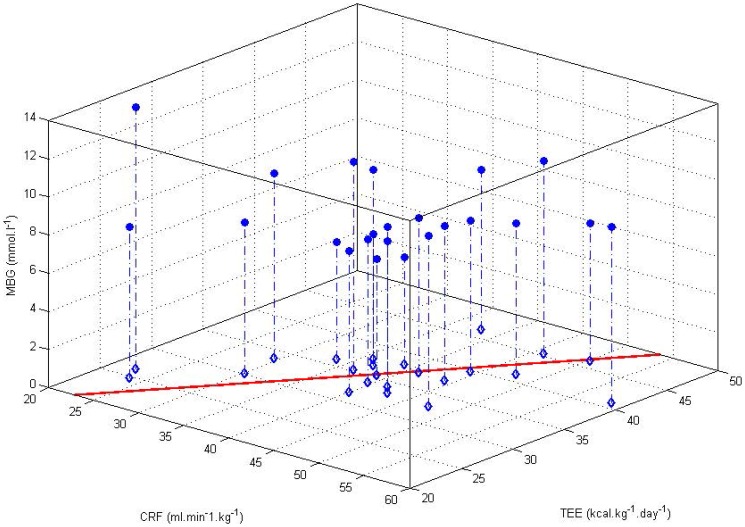
3-D Scatter plot for cardiorespiratory fitness (CRF), mean daily total energy expenditure (TEE) and mean blood glucose (MBG) of study participants. Blue diamonds show the relationship between CRF and TEE with the solid red line showing the linear fit (CRF  =  TEE + 2.3, R^2^  =  0.47). Blue circle markers show the corresponding MBG for each individual.

We examined further the relationships between CRF, TEE and MBG concentrations. The 3-D scatter plot in Figure 2 shows the relationship between CRF and TEE with the corresponding MBG for the participants in this study. At the extremes, relatively unfit and inactive individuals show markedly different MBG while similar albeit smaller variations are observable in the fitter and more active participants.

## Discussion

**Figure 3:**
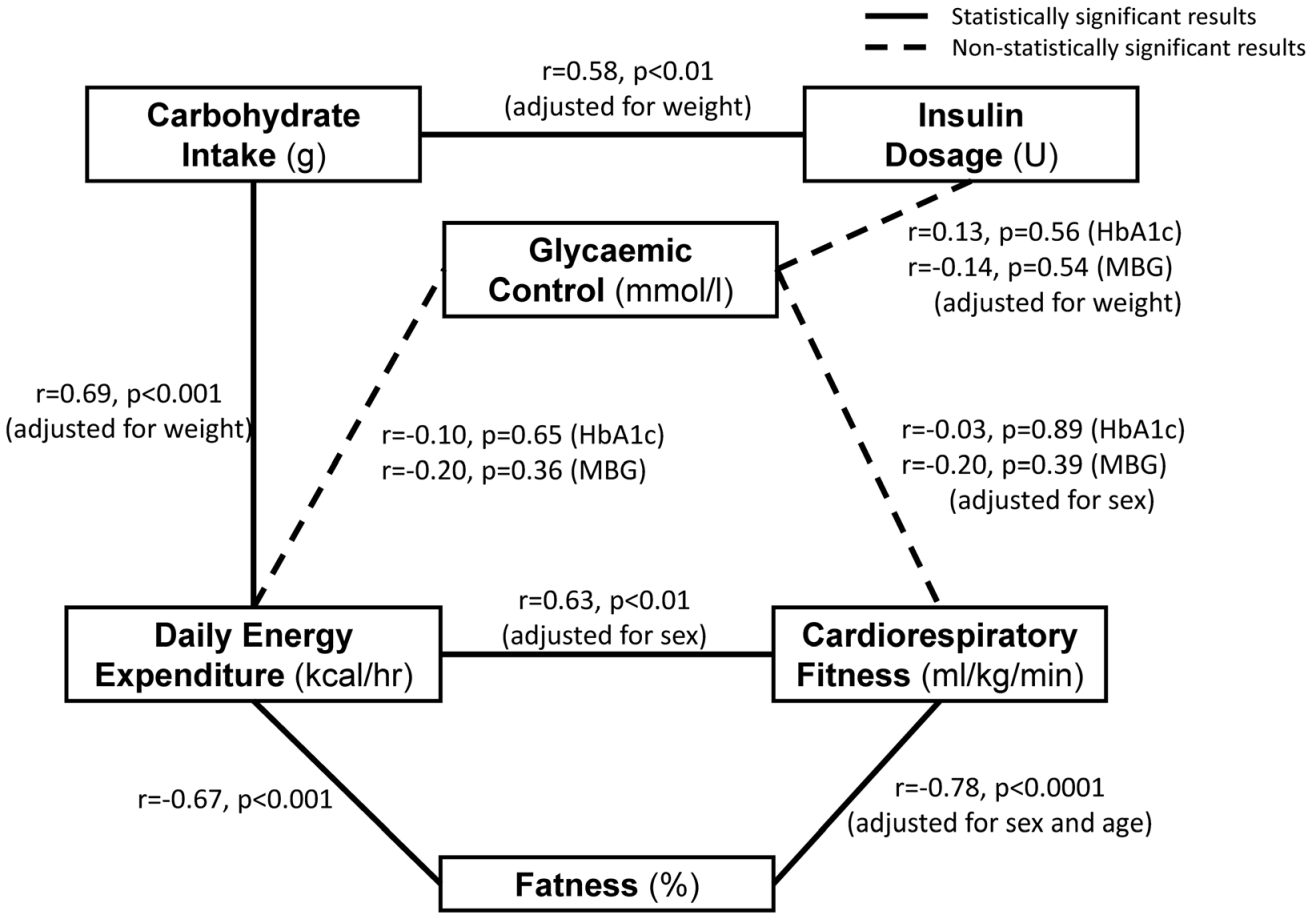
Summary of key findings describing the triangular relationship between glycaemic control, daily energy expenditure and cardiorespiratory fitness.

In this observational pilot study of a cohort of people with type 1 diabetes of different ages and lifestyles, we found that: a) daily energy expenditure was negatively associated with total body fat and positively correlated with CRF and mean carbohydrate intake; b) daily energy expenditure was not strongly correlated with the various measures of glycaemia, and in our relatively small data-set these correlations were not statistically significant. Figure 3 summarises pictorially the main findings of our study.

The main strength of our study was the use of an objective quantitative measure of energy expenditure, when compared to subjective lifestyle questionnaires, and the use of CGMS data captured over 8±3 days, (mean±SD) to obtain typical minute-by-minute variability in each individual's glycaemic control. The SenseWear Pro2/3 physical activity armband has been found to underestimate energy expenditure by 117 kcal day^−1^ [23], which is only about 5% of the recommended calorific intake of an average adult man. Whilst CGMS has been shown to have a consistent range-dependent bias, with overestimation of glucose concentrations within the hypoglycaemic range, no bias in the normoglycaemic range, and underestimation during hyperglycaemia [27].

No exercise intervention was prescribed for our volunteers, who were monitored in a free-living environment. Consequently the data captures routine behaviour for the people in our cohort. Although we acknowledge that the size of the cohort in our detailed physiological study has limited the power of the statistical methods used to detect associations, it is important to stress that associations between all of the measures of glycaemic control and physical activity were weak. The largest r-value computed was 0.2, which represents a small effect size if this association is real. A retrospective sample size calculation shows that we would have needed a cohort of 194 people to show statistical significance at the 0.05 level with 80% power to detect this effect.

We have shown that levels of daily energy expenditure were associated with body fat and CRF. Ekelund *et al.* [28] reported an inverse relationship between physical activity energy expenditure, estimated from heart rate monitoring, and fat mass in a large middle-aged healthy group. Similar results were reported by den Hoed and Westerterp [29] who found an association between body composition and physical activity in a study of 134 healthy individuals, measured by a triaxial accelerometer. In a healthy, but non-athlete group of thirty-eight people, habitual physical activity was associated with mitochondrial capacity [30]; this could have contributed for the association with CRF found in our group. CRF was found to be positively associated with both duration and intensity of incidental physical activity in a cohort of inactive and abdominally obese adults [31]. In a large longitudinal study of healthy individuals, Lakoski *et al.* [32] reported that 56% of the variance in CRF was explained by age, gender, BMI and physical activity, the latter being quantified by self-reported questionnaires. Our multiple regression results, (acknowledging our limited sample size), showed striking similarity with data from Lakoski *et al.* [32]. In our study, we showed that 56% of the variance in CRF in individuals with type 1 diabetes was explained by TEE and percentage total body fat, and we found that age did not contribute to this relationship. The normalised β-coefficients for TEE (β =  0.41) and percentage body fat (β =  −0.40) suggest that these modifiable factors have similar weights on the relationship with CRF, albeit in opposite directions. The difference between daily energy expenditure and CRF in their contribution towards health and general wellbeing is still a topic of active research [33]. The cardio-protective effects of physical activity are however undisputed [34] and recent results from a large study on adults aged over 60 years have shown a strong inverse correlation between physical fitness and all-cause mortality independent of fat distribution [35].

In our study group, carbohydrate intake was positively correlated with daily energy expenditure when adjusting for body weight. The positive correlation could be in part possibly explained by fear of hypoglycaemia [3] in those individuals who have a more active lifestyle, and possibly confounding the relationship between mean glycaemia and energy expenditure. There was a strong negative association between carbohydrate intake and sedentary levels of energy expenditure and a strong positive association with moderate levels of energy expenditure. This may suggest that more active individuals consume more carbohydrate and that in itself potentially causes some difficulties in maintaining glycaemic control.

**Figure 4:**
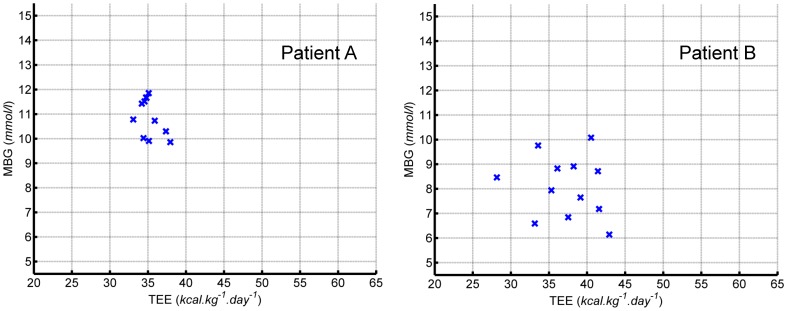
Plots for daily mean blood glucose (MBG) against daily total energy expenditure (TEE) for two individuals (every blue cross represents a new day). These two participants were chosen to illustrate the two extremes (amongst participants) in the relationships between daily MGB and TEE. Participant A showed very little day to day variability (CV TEE  =  0.04, MBG  =  0.07), whereas in contrast, Participant B showed much greater variability (CV TEE  =  0.12, MBG  =  0.15).

The majority of previous studies have tested the effect of specific lifestyle intervention programmes on long-term glycaemic control quantified by HbA_1C._ The results from those studies have been contradictory, with a number of studies reporting no improvement in HbA_1C_ following the training program [6], [8]–[12], [14], [19], [35]–[37], while in others an association was found between physical activity and long-term glycaemic control [7], [13], [16], [17] as reported here although very weak. In a study by Wallymahmed *et al.* [38] increased CRF was even associated with increased HbA_1C_. Such inconsistent results across various studies suggest that a biologically plausible relationship between levels of energy expenditure and glycaemic control is confounded by multiple factors as reported in [22]. Day-to-day data from CGMS and the physical activity armband allow us to shed some light on such factors, which could have confounded the relationship. Figure 4 shows a plot of daily blood glucose and energy expenditure for two individuals chosen to illustrate the two extremes (amongst participants) in the relationships between daily MGB and TEE. As can be seen individual A (HbA_1C_, 8.0%: MBG 11.0 mmol/l) exhibits less intra-individual variability than individual B (HbA_1C_, 7.6%: MBG 7.9 mmol/l). Intra-subject variability adds ambiguity to the overall analysis and interpretation of results, which is primarily aimed at explaining the inter-subject variation. Averaging data to characterise an individual's quality of glycaemic control and lifestyle obscures within day and between day changes in both variables. It is therefore debatable how to quantify the *typical* lifestyle and glycaemic control. HbA_1c_ is the *de facto* mean which clinicians use to assess their patients overall glycaemic control, and this was not found to be statistically significantly or strongly associated with MBG. Although the HbA_1c_ assay and CGMS measure different entities, glycated haemoglobin and interstitial glucose respectively, they should in theory return similar estimates for the average blood glucose concentrations. HbA_1c_ values for our cohort were fairly constant two years prior to being recruited in the study, with an average coefficient of variation (SD normalised by the mean, expressed as a percentage) of 6.2±3.5% (2.1 – 15.4%). One would have expected therefore that when observing the individuals for up to 12 days, the mean recorded by the CGMS would be strongly associated with HbA_1c._ There are however several other unaccounted factors that could have affected BG significantly in the short-term, such as psychological stress [39], [40], menstrual cycle [41] and the effect of the previous day, for example the initial state of glycogen depots in the liver and muscle.

## Conclusions

Our novel data show that in people with type 1 diabetes, daily energy expenditure is positively and strongly associated with cardiorespiratory fitness and cardiorespiratory fitness declines with diabetes duration. In contrast, the measures of daily energy expenditure were only weakly associated with several measures of glycaemic control. Our results suggest that people with type 1 diabetes who have a more active lifestyle exhibit both better cardiorespiratory fitness and less body fat, but not necessarily better glycaemic control. Since increased levels of energy expenditure and good glycaemic control are both needed to protect against diabetes-related complications and they are only weakly related, our data suggest they may need to be achieved independently as we have found no evidence of a strong relationship between energy expenditure and levels of glycaemia.

## Supporting Information

Checklist S1 **TREND checklist.**
(PDF)

Protocol S1 **Study protocol.**
(DOC)

## References

[1] Church TS, Cheng YL, Earnest CP, Barlow CE, Gibbons LW, et al (2004) Exercise capacity and body composition as predictors of mortality among men with diabetes. Diabetes Care 27(1): 83–88. 14693971 10.2337/diacare.27.1.83

[2] Church TS, LaMonte MJ, Barlow CE, Blair SN (2005) Cardiorespiratory fitness and body mass index as predictors of cardiovascular disease mortality among men with diabetes. Arch Intern Med 165(18): 2114–2120. 16217001 10.1001/archinte.165.18.2114

[3] McAuley P, Myers J, Emerson B, Oliveira RB, Blue CL, et al (2009) Cardiorespiratory fitness and mortality in diabetic men with and without cardiovascular disease. Diabetes Res Clin Pract 85(3): 30–33. 19524317 10.1016/j.diabres.2009.05.012

[4] Wild D, von Maltzahn R, Brohan E, Christensen T, Clauson P, et al (2007) A cricitical review of the literature on fear of hypoglycaemia in diabetes: Implications for diabetes management and patient education. Patient Educ Couns 68: 10–15. 17582726 10.1016/j.pec.2007.05.003

[5] Brazeau AS (2008) Rabasa-Lhoret R (2008) Strychar I (2008) Mircescu H (2008) Barriers to physical activity among patients with type 1 diabetes. Diabetes Care 31(11): 2108–2109. 18689694 10.2337/dc08-0720 PMC2571055

[6] The Diabetes Control and Complications Trial Research Group (1993) The effect of intensive treatment of diabetes on the development and progression of long-term complications in insulin-dependent diabetes mellitus. New Engl J Med 329: 977–985. 8366922 10.1056/NEJM199309303291401

[7] Lehmann R, Kaplan V, Bingisser R, Bloch KE, Spinas GA (1997) Impact of physical activity on cardiovascular risk factors in IDDM. Diabetes Care 20(10): 1603–1611. 9314643 10.2337/diacare.20.10.1603

[8] Mosher PE, Nash MS, Perry AC, LaPerriere AR, Goldberg RB (1998) Aerobic circuit exercise training: effect on adolescents with well-controlled insulin-dependent diabetes mellitus. Arch Phys Med Rehab 79(6): 652–657. 10.1016/s0003-9993(98)90039-9 9630144

[9] Rigla M, Sanchez-Quesada JL, Ordonez-Llanos J, Prat T, Caixas A, et al (2000) Effect of physical exercise on lipoprotein(a) and low density lipoprotein modifications in type 1 and type 2 diabetic patients. Metabolism 49(5): 640–647. 10831176 10.1016/s0026-0495(00)80041-4

[10] Laaksonen DE, Atalay M, Niskanen LK, Mustonen J, Sen CK, et al (2000) Aerobic exercise and the lipid profile in type 1 diabetic men: a randomized controlled trial. Med. Sci. Sports Exerc 32(9): 1541–1548. 10.1097/00005768-200009000-00003 10994902

[11] Wiesinger G, Pleiner J, Quittan M, Fuchsjager-Mayrl G, Crevenna R, et al (2001) Health related quality of life in patients with long-standing insulin dependent (type 1) diabetes mellitus: benefits of regular physical training. Wien Klin Wochenschr 113: 670–675. 11603101

[12] Roberts L, Jones TW, Fournier PA (2002) Exercise training and glycemic control in adolescents with poorly controlled type 1 diabetes mellitus. JPediatr Endocr Met 15(5): 621–627. 10.1515/jpem.2002.15.5.621 12014521

[13] Fuchsjager-Mayrl G, Pleiner J, Wiesinger GF, Sieder AE, Quittan M, et al (2002) Exercise training improves vascular endothelial function in patients with type 1 diabetes. Diabetes Care 25(10): 1795–1801. 12351480 10.2337/diacare.25.10.1795

[14] Michaliszyn SF, Faulkner MS (2010) Physical activity and sedentary behaviour in adolescents with type 1 diabetes. Res Nurs Health 33(5): 441–449. 20672318 10.1002/nur.20393 PMC4354709

[15] Mittermayer F, Pleiner J, Krzyzanowska K, Wiesinger GF, Francesconi M, et al (2005) Regular physical exercise normalizes elevated asymmetrical dimenthylarginine concentrations in patients with type 1 diabetes mellitus. Wien Klin Wochenschr 117: 816–820. 16437318 10.1007/s00508-005-0476-y

[16] Kavookjian J, Elswick BM, Whetsel T (2007) Interventions for being active among individuals with diabetes: A systematic review of the literature. Diabetes Educator 33(6): 962–988. 18057265 10.1177/0145721707308411

[17] Herbst A, Bachran R, Kapellen T, Holl RW (2006) Effects of regular physical activity on control of glycemia in pediatric patients with type 1 diabetes mellitus. Arch Pediat Adol Med 160(6): 573–577. 10.1001/archpedi.160.6.573 16754817

[18] Benevento D, Bizzarri C, Pitocco D, Crino A, Moretti C, et al (2010) Computer use, free time activities and metabolic control in patients with type 1 diabetes. Diabetes Res Clin Pr 88(3): 32–34. 10.1016/j.diabres.2010.03.016 20378196

[19] Waden J, Tikkanen H, Forsblom C, Fagerudd J, Pettersson-Fernholm K, et al (2005) Leisure time physical activity is associated with poor glycemic control in type 1 diabetic women: the FinnDiane study. Diabetes Care 28(4): 777–782. 15793172 10.2337/diacare.28.4.777

[20] Ligtenberg PC, Blans M, Hoekstra JB, van der Tweel I, Erkelens DW (1999) No effect of long-term physical activity on the glycemic control in type 1 diabetes patients: a cross-sectional study. Neth J Med 55(2): 59–63. 10474273 10.1016/s0300-2977(99)00039-x

[21] Wallymahmed M, Morgan C, Gill G, MacFarlane I (2007) Aerobic fitness and hand grip strength in type 1 diabetes: relationship to glycaemic control and body composition. Diabetic Med 24(11): 1296–1299. 17956456 10.1111/j.1464-5491.2007.02257.x

[22] Bonnefoy M, Normand S, Pachiaudi C, Lacour JR, Laville M, et al (2001) Simultaneous validation of ten physical activity questionnaires in older men: A doubly labeled water study. J Am Geriatr Soc 49: 28–35. 11207839 10.1046/j.1532-5415.2001.49006.x

[23] St-Onge M, Mignault D, Allison DB, Rabasa-Lhoret R (2007) Evaluation of a portable device to measure daily energy expenditure in free-living adults. Am J Clin Nutr 85: 742–749. 17344495 10.1093/ajcn/85.3.742

[24] Church TS, Thomas DM, Tudor-Locke C, Katzmarzyk PT, Earnest CP, et al (2011) Trends over 5 Decades in U.S. Occupation-Related Physical Activity and Their Associations with Obesity. PLoS ONE 6(5): e19657 doi:10.1371/journal.pone.0019657 21647427 10.1371/journal.pone.0019657 PMC3102055

[25] Foster C, Jackson AS, Pollock ML, Taylor MM, Hare J, et al (1984) Generalized equations for predicting functional capacity from treadmill performance. Am Heart J 107: 1229–1234. 6720550 10.1016/0002-8703(84)90282-5

[26] Pollock ML, Foster C, Schmidt D, Hellman C, Linnerud AC, et al (1982) Comparative analysis of physiologic responses to 3 different maximal graded-exercise test protocols in healthy women. Am Heart J 103: 363–373. 7064770 10.1016/0002-8703(82)90275-7

[27] Gross TM, Bode BW, Einhorn D, Kayne DM, Reed JH, et al (2000) Performance evaluation of the MiniMed continuous glucose monitoring system during patient home use. Diabetes Technol Ter 2(1): 49–56. 10.1089/152091500316737 11467320

[28] Ekelund U, Brage S, Franks PW, Hennings S, Emms S, et al (2005) Physical activity energy expenditure predicts changes in body composition in middle-aged healthy whites: effect modification by age. Am J Clin Nutr 81(5): 964–969. 15883416 10.1093/ajcn/81.5.964

[29] den Hoed M, Westerterp KR (2008) Body composition is associated with physical activity in daily life as measured using a triaxial accelerometer in both men and women. Int J Obesity 32: 1264–1270. 10.1038/ijo.2008.72 18504444

[30] den Hoed M, Hesselink M, van Kranenburg G, Westerterp KR (2008) Habitual physical activity in daily life correlates positively with markers for mitochondrial capacity. J Apply Physiol 105(2): 561–568. 10.1152/japplphysiol.00091.2008 18511526

[31] McGuire KA, Ross R (2011) Incidental physical activity is positively associated with cardiorespiratory fitness. Med. Sci. Sports Exerc 43(11): 2189–94. 10.1249/MSS.0b013e31821e4ff2 21502894

[32] Lakoski SG, Barlow CE, Farrell SW, Berry JD, Morrow JR, et al (2011) Impact of body mass index, physical activity, and other clinical factors on cardiorespiratory fitness (from the Cooper Center Longitudinal Study). Am J Cardiol 108(1): 34–39. 21529738 10.1016/j.amjcard.2011.02.338

[33] Blair S, Cheng Y, Holder J (2001) Is physical activity or physical fitness more important in defining health benefitts? Med. Sci. Sports Exerc 33: S379–S399. 11427763 10.1097/00005768-200106001-00007

[34] Archer E, Blair SN (2011) Physical activity and the prevention of cardiovascular disease: From evolution to epidemiology. Prog Cardiovasc Dis 55: 387–396. 10.1016/j.pcad.2011.02.006 21545924

[35] Sui X, LaMonte MJ, Laditka JN, Hardin JW, Chase N, et al (2007) Cardiorespiratory fitness and adiposity as mortality predictors in older adults. JAMA 298(21): 2507–2516. 18056904 10.1001/jama.298.21.2507 PMC2692959

[36] Ramalho AC (2006) de Lourdes Lima M (2006) Nunes F, Cambui Z, Barbosa C, et al (2006) The effect of resistance versus aerobic training on metabolic control in patients with type-1 diabetes mellitus. Diabetes Res Clin Pr 72(3): 271–276. 10.1016/j.diabres.2005.11.011 16406128

[37] Harmer AR (2007) Chisholm DJ (2007) McKenna MJ, Morris NR, Thom JM, et al (2007) High-intensity training improves plasma glucose and acid-base regulation during intermittent maximal exercise in type 1 diabetes. Diabetes Care 30 ((5)) 1269: 1271. 10.2337/dc06-1790 17325264

[38] Wallymahmed M, Morgan C, Gill G, MacFarlane I (2007) Does hypoglycaemic avoidance behaviour contribute to increased hba1c levels in physically active people with type 1 diabetes? Practical Diabetes Int 24(8): 418–421.

[39] Riazi A, Pickup J, Bradley C (2004) Daily stress and glycaemic control in Type 1 diabetes: individual differences in magnitude, direction, and timing of stress-reactivity. Diabetes Res Clin Pr 66 ((3)): 237–244. 10.1016/j.diabres.2004.04.001 15536020

[40] Wiesli P, Klaghofer R, Schmid C (2005) Acute psychological stress affects glucose concentrations in patients with type 1 diabetes following food intake but not in the fasting state. Diabetes Care 28: 1910–1915. 16043731 10.2337/diacare.28.8.1910

[41] Trout KK, Rickels MR, Schutta MH, Petrova M, Freeman EW, et al (2007) Menstrual cycle effects on insulin sensitivity in women with type 1 diabetes: a pilot study. Diabetes Technol Ter 9(2): 176–182. 10.1089/dia.2006.0004 17425444

