# Supplementary material for: Daily Energy Expenditure, Cardiorespiratory Fitness and Glycaemic Control in People with Type 1 Diabetes
Source: PLoS One. 2014 May 14;9(5):e97534. doi: 10.1371/journal.pone.0097534 (PMC4020853; doi:10.1371/journal.pone.0097534)
Supplement: Protocol S1 — Study protocol. (DOC) [file pone.0097534.s002.doc]

**Protocol**

**Title of project**

**Dynamic modelling of the effect of physical activity on capillary blood glucose concentration.**

**Diabetes UK awarded, peer reviewed and funded PhD Studentship 3 years starting Autumn 2007**

**Principal applicant**

Christopher D Byrne FRCP FRCPath PhD
Professor Endocrinology & Metabolism
Institute of Developmental Sciences
(University of Southampton)
Southampton General Hospital (MP887)
Tremona Road
Southampton. SO166YD
tel: 44 (0)23 80798818
fax: 44 (0)23 80795255
email: [cdtb@soton.ac.uk](mailto:cdtb@soton.ac.uk)

[www.metabolicsyndrome.org.uk](blocked::http://www.metabolicsyndrome.org.uk)
[http://www.som.soton.ac.uk/research/dohad/](blocked::http://www.som.soton.ac.uk/research/dohad/)

PA: Lucinda England
Tel: 44 (0)23 80 795006
Email: [L.C.England@soton.ac.uk](mailto:L.C.England@soton.ac.uk)

**Co-applicants**

Dr AJ Chipperfield

Senior Lecturer in Computational Methods

School of Engineering Sciences

University of Southampton

Highfield

Southampton. SO17 1BJ.

[A.J.Chipperfield@soton.ac.uk](mailto:A.J.Chipperfield@soton.ac.uk)

Tel: 02380 59 8344

Fax: 02380 59 4813

Geraldine F Clough BSc PhD

Professor of Vascular Physiology

Institute of Developmental Sciences

(IDS Building)

University of Southampton

Southampton General Hospital

MP 887, Southampton SO16 6YD. UK

email [g.f.clough@soton.ac.uk](mailto:g.f.clough@soton.ac.uk)

Tel: 02380 794292

**Summary**

At present it is uncertain how day to day variation in physical activity levels, influence blood glucose in people with type 1 diabetes. We can now study this scientific question because sophisticated light, user-friendly physical activity monitoring devices have been manufactured and are licensed and validated for measuring physical activity energy expenditure. We will lend volunteers a device which they wear around their upper arm like a watch with an elasticated band. Volunteers can sleep wearing the device and this device allows us to monitor the amount of energy burnt up through physical activity levels. We will then precisely assess physical activity levels in relation to capillary blood glucose levels. People with type 1 diabetes measure their capillary blood glucose concentrations routinely as part of their monitoring glucose control. In addition to this we will also lend volunteers a continuous blood glucose monitoring device (Guardian® Real-Time System) which records continuously the blood glucose levels throughout the day. This sophisticated device is minimally invasive and virtually painless. Our study has been subjected to scientific peer review and in 2007 has been approved and funded by the national diabetes charity

Diabetes UK.

We plan to issue each participant a SenseWear Pro physical activity monitoring device compatible with their personal blood glucose meter and instructions for its operation. This will ideally occur at the same time as their HbA1c and BMI are measured at clinical review. Recording of patient medication, date of diagnosis, alcohol consumption, smoking status, and other personal parameters will be assessed by questionnaire. Participants will also be asked to record their insulin treatment patterns. We will also assess food intake by ‘recent re-call of food consumption’ (asking participants to keep a diary of their food intake) to tell us what they are eating each day during this period.

The SenseWear Pro physical activity armband is capable of recording up to one month’s continuous data. The data can be retrieved by physically returning the device, e-mail transfer or via a secure web-based system.

Also for a period of time (1 week, if possible two weeks) during which the patient is wearing the SenseWear armband, we will lend the patient a continuous blood glucose monitoring device (Guardian® Real-Time System), which will record and store continuously the blood glucose levels throughout the day.

Data will be captured from subjects twice over periods of up to a month, with a six month interval between observations. A target of 6 to 8 days per month is required for the short term modelling activity (but this is, of course, dependent on the characteristics and dynamics of the individual subject and cannot be determined precisely *a priori*). The one month period allows bad or corrupted data subsets to be identified and ensures that external and environmental parameters that induce variations in response are adequately sampled. It will also ensure that there is sufficiently rich data to design and validate models. The six month interval between samples will be used to analyse the seasonal variation in activity, diet and other observed factors as well as for identifying the parameters of long term trends using the techniques described below.

Clearly, even in the small pilot study proposed here, large quantities of data will be produced and require careful management. Techniques developed for grid computing in the Southampton eScience Centre (within Dr Chipperfield’s research group) will be harnessed to manage the data sets and automate the search for successful models. Use will be made of the new high performance computing cluster recently installed at the eScience Centre to allow a wider range of model classes and structures to be evaluated than would be possible with conventional PCs. The classes of models considered will include compartmentalised models with adaptation for energy utilisation described previously, as these represent a benchmark in current practice. More recent data-true model classes, including nonlinear state space [1] and Volterra models [2] have been shown to be effective in this respect particularly in their ability to deal with noisy and/or missing data but have not been applied in this context. While no particular technique is advocated *a priori*, this research will seek to devise pragmatic models that are demonstrably effective following the general inductive, data-true approach advocated by Marmarelis [3]. Models will be validated against data sets, or portions thereof, that are not used to identify the model and assessed for their clinical and scientific relevance. To help ensure statistical relevance, models from individual data-sets and their associated parameters will be analysed against one another to assess their general validity and parametric variance.

**Specific aims of proposed investigation**

The primary aim of this research is to develop data-true models, from an observational pilot study, that characterize an individual’s pattern of physical activity and corresponding capillary blood glucose control both in the short and long term. In particular, this research will:

1. Investigate and describe the relationship between lifestyle and quality of glycaemic control using patient-acquired data for physical activity and blood glucose concentration;
2. Determine classes of data-true mathematical models of the response of blood glucose concentration to physical activity in the short term, i.e. daily or weekly, that accurately characterize specific individuals; and
3. Identify the parameters of such models that vary in the longer term, i.e. over months or seasons, which characterize the variations in an individual diabetic person’s glycaemic control.

**Detailed** **background of the project**

The population of Southampton is 221,200 with a prevalence of known type 1 and type 2 diabetes of 3.01%. Assuming prevalence of type 1 diabetes of 0.51% there is a target population of 1128 people for this pilot study. We plan to initially invite ~60 people to participate - men and women aged 18-75 years, from the diabetes clinics, at Southampton University Hospitals Trust, covering a range of BMI, and level of physical activity. Assuming a response rate of 50% this will provide a set of ~30 exemplars. The specific number of invitations will be adapted as required to ensure that a representative sample of the local population and its diversity is obtained. It is anticipated that these subjects will be recruited from the patient registers of people with type 1 diabetes attending the diabetes clinics at Southampton University Hospitals Trust where Professor Byrne works as a Consultant Diabetologist.

To help ensure general validity of the study, subjects with other known pre-existing conditions will be excluded.

A significant proportion of people with type 1 diabetes suffer from unsatisfactory glycaemic regulation. Improving an individual’s understanding of the lifestyle parameters, particularly the role of physical activity is expected to be of benefit in ameliorating the complications of diabetes. Given the increasing prevalence of obesity in the general population, techniques such as those proposed here will be necessary if the special problems facing people with type 1 diabetes are to be effectively addressed.

Identifying and promoting lifestyle modifications that encourage physical activity is a fundamental part of the treatment of type 1 diabetes. Indeed, regular physical exercise is encouraged as, in general, it lowers glucose concentration during and after exercise and increases insulin use by cells. The consequent benefit through improved glycaemic control is the delay, or avoidance, of long-term complications such as retinopathy and peripheral neuropathy. As type 1 diabetics are usually young and physically active at diagnosis, establishing a pattern of insulin, diet and exercise that achieves good blood glucose control with a low risk of hypo- and hyperglycaemia is vital for long-term health as well as general wellbeing. However, there is no simple formulaic approach to finding such a balance between these parameters and each individual will experience many variations in them over a given period of time. Understanding the relationships between these three main control parameters and glycaemic control is of great significance. Data-true models of individual patients that characterize these relationships may be used in a number of beneficial ways from patient and clinical training to understanding the physiological basis for observed behaviour or designing personalised care programmes.

There have been many studies that have tried to model the dynamics of insulin and glucose. Traditional modelling approaches use compartmentalized models and, typically, a principle dynamic mode analysis to determine the parameters of a physiology-based model within the individual compartments [4]. The subject data is used to fit parameters to the model, (e.g., for glucose plasma concentration or insulin action) minimizing some error or performance criteria (e.g., one-step-ahead prediction error or minimum residual variance). The majority of these studies use the variation of insulin doses as the primary control variable with meals and physical activity considered as disturbances. The most successful applications of these models has been in closed loop insulin control in intensive care situations where diet and activity remain constant and controlled and measurements of capillary blood glucose concentration are continually available (see, for example, [2]. There are a number of problems associated with this approach. The differential equations representing the essential glucose metabolism and insulin secretion do not fully capture the nonlinear and time varying nature of the underlying dynamics or other processes such as independent glucose utilization. Models derived from measurement data obtained in a clinical setting are often unsuited to modelling the daily life of the diabetic person, can exhibit large errors and are only suited to short-term characterization of an individual. For these reasons some more recent studies, e.g. [5], have started to develop methods that attempt to account for carbohydrate intake and/or energy expenditure but so far these have been limited to generic studies using artificial data sets.

The level of physical activity, its intensity and duration will exhibit considerable variation over a period of time and, until recently, has been difficult to parametrically quantify in a meaningful way. The use of expensive metabolic carts can be cumbersome and is unsuited to long-term usage although high quality patient measurements can be obtained. Accelerometers and pedometers offer a more practical method of patient data acquisition although they yield far less reliable results with many sources of error arising from the conversion of raw measurements and the approximation of energy usage. Additionally, data collected from patient administered blood tests may not synchronize with that for physical activity and may thus introduce further errors or bias into any analysis or models constructed from such data.

New wearable devices, such as BodyMedias’ SenseWear body monitoring system have previously been shown to be an effective method of capturing the energy expenditure in free-living adults with diabetes [6]. Using a combination of regular measurements of skin temperature, Galvanic skin response, heat flux and two-axis accelerometers this unobtrusive 82 gram device is capable of accurately monitoring total and active energy expenditure, METs, number of steps, physical activity and sleep duration as well as determining when the subject is lying down. It is suitable for wearing under clothing and can be worn continuously with no discomfort. Outside of the clinical setting, these devices allow a richer and more diverse ensemble of patient data to be captured to compliment that normally available. Additionally, as the SenseWear device is data compatible with Roche glucose meters patient-data synchronization is achieved automatically as well as simplifying data management. The extra richness and fidelity in the obtainable data can then be harnessed to derive models that will more accurately represent the individual from whom the data was obtained.

Suitable models for characterizing the relationships between an individual’s activity profile and quality of glycaemic control must be dynamic as blood glucose levels are dependant on previous states of the system as well as the current ones along with its inputs. The model must also be adaptive as the human and exogenous parameters will vary over time as will the strength of interactions between the underlying processes. As a result, a number of different candidate approaches have been identified that offer improved predictive capabilities as well as fidelity to the modelled data and parameters. These include both deductive physics-based and inductive data-true model types.

Given the multidisciplinary nature of the proposed research programme, the supervision requirements will vary depending on the task being undertaken, the expertise required and the level of supervision necessary. The day-to-day supervision will be undertaken by Professor Byrne and Dr Chipperfield and Professor Clough.

**Volunteers will be recruited from the Diabetes Resource Centre data-base, at Southampton University Hospitals Trust.**

Professor Byrne has access to this data-base as a Consultant Diabetologist at Southampton University Hospitals Trust. Professor Byrne will invite people by letter of invitation with type 1 diabetes registered on the data-base to participate in the study. Professor Clough (co-applicant) and Dr Chipperfield (co-applicant) and John Valletta (PhD student working on the project) will also have access to the database.

**Visit Description List**

**Visit 1 *Clinical Tests***

VO_2_ max (fitness test)

Indirect calorimetry (test of metabolic rate)

Hand grip strength (test of muscle strength)

Ankle-Brachial pressure index (test of peripheral blood flow)

Blood test (kidney, liver and thyroid function, HbA_1_C and cholesterol levels)

Microalbuminiria (test of protein excretion in urine)

Body composition using bioimpedance, DEXA scan and Bod Pod

(assessment of body fat)

Microvascular function and muscle/skin blood flow (assessment of muscle

and skin blood flow)

(Some of these tests can be undertaken on visit 2)

***Volunteer’s Tasks***

Wear the SenseWear^TM^ physical activity monitoring device for a minimum of

one week up to two weeks (depending on the volunteer’s availability)

Record their blood glucose levels when pricking their fingers

Record their daily insulin dosage, as part of their normal clinical care.

Record their daily food intake

**Visit 2** This visit will take place one to two weeks after visit 1

***No Clinical Tests***

***Collect the data***

***Volunteer’s Tasks***

Same as visit 1 but the volunteer will now be given a continuous blood

glucose measuring device (Guardian® Real-Time System) which has to be worn for a minimum of one week up to two weeks (depending on the volunteer’s availability)

**Visit 3** This visit will take place one to two weeks after visit 2

***No Clinical Tests***

***Collect the Data***

***No Volunteer’s Tasks – End of first part of study***

**Visit 4** This visit will take place four to six months after visit 3

***No Clinical Tests***

***Volunteer’s Tasks***

Same as visit 1

**Visit 5** This visit will take place one to two weeks after visit 4

***No Clinical Tests***

***Collect the Data***

***Volunteer’s Tasks***

Same as visit 2

**Visit 6** This visit will take place one to two weeks after visit 5

***Clinical Tests***

Blood test (kidney, liver and thyroid function, HbA_1_C and cholesterol levels)

Indirect calorimetry (test of metabolic rate)

Body composition using bioimpedance, DEXA scan and Bod Pod

(assessment of body fat)

***Collect the Data***

***No Volunteer’s Tasks – End of Study***

**Appendix**

**Additional information – Clinical Tests**

**Microvascular measurements (leg blood flow)**

- This will be measured using a non-invasive and painless technique, that have no known effects on skin or muscle:

Whilst lying flat, an elastic gauge will be placed around your calf and a cuff will be placed around your thigh. In addition two low-intensity laser probes (skin and muscle probe) will be attached to the skin of your calf. Small increases in pressure will then be applied via the cuff and maintained. Each step will last approximately 4 minutes. The cuff will then be rapidly inflated for 3 minutes. The whole process will take no longer than 1 hour.

**Measurement of peripheral circulation**

- *Ankle / Brachial Pressure Index (ABI)* - is a simple measurement for assessing peripheral macrovascular function (blood flow in legs). Low ABI (<0.9) is an independent predictor of increased cardiovascular disease risk. We want to assess relationships between ABI and other measures such as physical activity levels and fitness. With you rested and lying flat on your back, blood pressure cuffs will be placed bilaterally on your upper arm (brachial pressure) and ankle and then inflated. An ultrasound probe will be placed over your arm and leg arteries in turn. This is a painless and very safe procedure.

**Measurement of muscle strength**

- *Handgrip strength –* We also wish to examine the relationship between how active you are and how strong your muscles are. We can do this simply by asking you to squeeze a small measuring device that you hold in your hand (a Jamar dynamometer). We will use for the analysis the best score out of the total three measurements from each hand. The Jamar dynamometer is simple to use, accurate, reproducible in its measurements and the most widely used and reported upon devices assessing muscle strength.

**Measurement of fitness**

- *VO_2_ max* – By breathing into a mask while exercising on a treadmill, we can measure the amount of oxygen you breathe in and how much carbon dioxide you breathe out. We will gradually increase the difficulty of the exercise until the maximal oxygen consumption is recorded (VO_2_max). People with type 1 diabetes are usually young and physically active; this will give us an indication of the fitness level of each and every volunteer which we can then relate to their glycaemic control.

**Measurement of metabolic rate**

- *Indirect calorimetry* – By breathing into a mask while you are lying on a bed we are able to measure how much oxygen you breathe in and how much carbon dioxide you breathe out. From these measurements we can calculate the respiratory quotient (the ratio of the volume of carbon dioxide produced to the volume of oxygen consumed) and the resting energy expenditure (calories you ‘burn’ up at rest).

**Blood test**

- The usual blood test which is used to assess various conditions, specifically kidney, liver and thyroid functions, glycated haemoglobin (HbA_1_C - gives an indication of the glycaemic control during the last three months) and cholesterol levels (lipid profile).

**Measurement of protein excretion**

- *Microalbuminiria* – This test measures the levels of albumin (the most abundant protein in the blood plasma) in the urine. Microalbuminiria is a marker of increased risk of cardiovascular and kidney diseases.

**Measurement of body composition**

- *Bioimpedance* – A small current of fixed frequency is passed through the body. The recorded impedance (opposition to flow of current) can be used to estimate the amount of body fat.
- *DEXA Scan* – Uses two X-ray beams of different energy levels which scan the whole body. Depending on the X-ray’s absorption, an estimate of the amount of body fat can be obtained.
- *Bod Pod* – Calculates the body volume by measuring the volume of air displaced while the person is sitting inside a comfortable chamber. Using the volume and the mass of the person (measured by an electronic scale), the density can be calculated. This measurement is then used to calculate the proportion of fat and lean mass by using scientifically derived equations.

References

[1] Briegel T, Tresp V. A non linear state space model for the blood glucose metabolism of a diabetic. Automatisierungstechnik 2002; **50**(5)**:** 228-236.

[2] Florian JA, Parker RS. A non linear data-driven approach to type 1 diabetic patient modelling. Proc 15th IFAC World Congress . 2002.
Ref Type: Conference Proceeding

[3] Marmarelis V Z. Nonlinear dynamic modelling of physiological systems. New Jersey: 2004.

[4] Bergman RN, Ider YZ, Bowden CR, Cobelli C. Quantitative estimation of insulin sensitivity. Am J Physiol 1979; **236**(6)**:** E667-E677.

[5] Derouich M, Boutayeb A. The effect of physical exercise on the dynamics of glucose and insulin. J Biomech 2002; **35**(7)**:** 911-917.

[6] Mignault D, St-Onge M, Karelis AD, Allison DB, Rabasa-Lhoret R. Evaluation of the Portable HealthWear Armband: a device to measure total daily energy expenditure in free-living type 2 diabetic individuals. Diabetes Care 2005; **28**(1)**:** 225-227.
